# Factors that influence the way local communities respond to consultation processes about major service change: A qualitative study

**DOI:** 10.1016/j.healthpol.2015.04.015

**Published:** 2015-09

**Authors:** Helen Barratt, David A. Harrison, Rosalind Raine, Naomi J. Fulop

**Affiliations:** a Department of Applied Health Research, University College London, 1-19 Torrington Place, London WC1E 7HB, UK; b Intensive Care National Audit and Research Centre, 24 High Holborn, London WC1V 6AZ, UK

**Keywords:** Great Britain, Emergency departments, Hospital planning, Patient involvement, Qualitative research

## Abstract

**Objectives:**

In England, proposed service changes such as Emergency Department closures typically face local opposition. Consequently, public consultation exercises often involve protracted, hostile debates. This study examined a process aimed at engaging a community in decision-making about service reconfiguration, and the public response to this process.

**Methods:**

A documentary analysis was conducted to map consultation methods used in an urban area of England where plans to consolidate hospital services on fewer sites were under discussion. In-depth interviews (*n* = 20) were conducted with parents, older people, and patient representatives. The analysis combined inductive and deductive approaches, informed by risk communication theories.

**Results:**

The commissioners provided a large volume of information about the changes, alongside a programme of public events. However, the complexity of the process, together with what members of the public perceived to be the commissioners’ dismissal of their concerns, led the community to question their motivation. This was compounded by a widespread perception that the proposals were financially driven.

**Discussion:**

Government policy emphasises the importance of clinical leadership and ‘evidence’ in public consultation. However, an engagement process based on this approach fuelled hostility to the proposals. Policymakers should not assume communities can be persuaded to accommodate service change which may result in reduced access to care.

## Highlights

• In England, service change proposals often face public opposition.
• We examined the public response to a consultation process about service reorganisation.
• The behaviour of key decision-makers led the public to mistrust the process.
• This was compounded by the complexity of the consultation methods.
• Future consultations should acknowledge public concerns about proposals.

## 1 Introduction

Health care systems around the world face the challenge of meeting rising demand for care with diminishing financial resources [1,2]. Attempts to tackle this dilemma may involve reorganising health care, for example by consolidating services across a region on fewer hospital sites. In England, whilst decision-makers seek potential health gains for patients by reorganising care, as well as cost savings, service change proposals often face public opposition. This commonly centres on concerns about future access to services [3,4]. Plans to alter Emergency Department (ED) services typically create the greatest concern [5]. Much of the public anxiety relates to the safety of centralised services and the potential risks that may be involved in having to travel further for care in an emergency [5]. Communities often argue that ‘lives will be put at risk,’ if such proposals go ahead [1].

Consequently, risk is part of the national discourse about service reorganisation, or reconfiguration. In the UK, reconfiguration is defined as ‘a deliberately induced change of some significance in the distribution of medical, surgical, diagnostic and ancillary specialties that are available in each hospital or other secondary or tertiary acute care unit in locality, region or health care administrative area’ [6]. It is a measure of change that directly addresses operational rather than structural change: hospitals may merge, form networks, or change their divisional or governance structures, without reconfiguring services. Spurgeon et al. point to parallels between the reconfiguration process and the literature about technological or environmental risks. Public perceptions about the risks involved in service reconfiguration are frequently at odds with the view put forward by ‘expert’ decision-makers, as is often the case with environmental hazards. They suggest that this may be because proponents and opponents of change operate within different paradigms of understanding about risk [4]. Sociologist Brian Wynne's research about the intersection of lay and expert knowledge offers a means of explaining this. Informed by the contextual model of risk communication, Wynne acknowledges that individuals do not simply respond as empty containers for information [7,8]. Instead, the way the public process risk information is shaped by their previous experiences and personal circumstances [9]. Wynne argues that the public are likely to be sceptical, critical or hostile to scientific statements when ‘expert’ accounts of physical reality conflict with their knowledge and understanding [10].

**Fig. 1:**
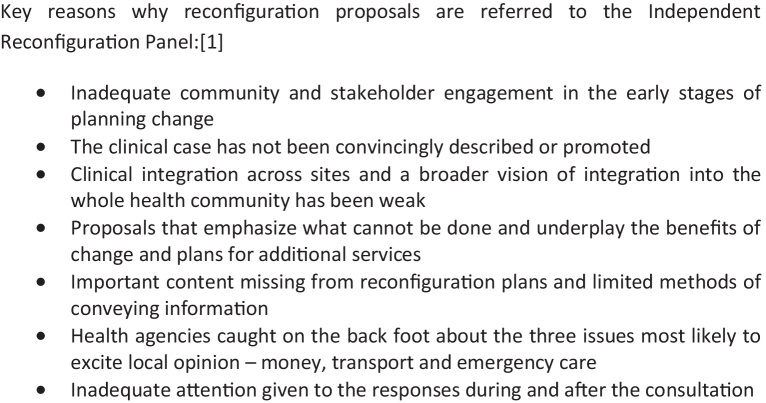
Key reasons why reconfiguration proposals are referred to the Independent Reconfiguration Panel.

In England, Section 242 of the NHS Act 2006 requires health care managers and purchasers – known as commissioners – to seek the views of affected parties, including patients and the public, if changes to local NHS services are being considered [11]. However, this consultation process often involves protracted, sometimes hostile local debates, leading to delays which some argue pose ‘significant risks to the delivery of safe services’ [12]. At the same time, there is a perception that the public do not in reality have an opportunity to influence the outcome of the decision-making process [5]. The Independent Reconfiguration Panel (IRP), provides the UK government with independent advice about reconfiguration proposals, when local agreement cannot be reached [1]. Local government representatives in affected areas may refer proposals to the Secretary of State for Health if they believe either that the consultation has been inadequate, or that the proposals are not in the best interest of the local population. The Secretary of State may then seek the advice of the IRP [12]. By mid-2012 the IRP had undertaken 19 full reviews of contested plans for health service change in England and offered written advice on several others [3]. The most frequent reasons for referral to the IRP are listed in Fig. 1 [1].

In light of these concerns, several groups have called for improvements in both the policy and process of public consultation about proposed service reorganisations [5,12]. To address this, government documents increasingly emphasise the role of ‘evidence’ and better consultation with the public, apparently assuming that if local communities are ‘involved enough’ and are presented with the ‘right evidence’ they will be convinced of the need to change [6]. However, Wynne and others have repeatedly shown that efforts to ‘educate’ the public by decreasing ‘deficits’ in their understanding typically fail because the ‘expert’ view conflicts with local people's knowledge and understanding [4,13].

A limited literature examines the process of reconfiguring hospital services, especially the dynamics of local decision-making [5]. Two previous studies explored the views of a range of individuals engaged in reorganisation [5,6], but neither examined the public engagement process in detail. This paper presents the findings of a qualitative study examining the process of public engagement in an urban area where major changes to hospital emergency services were being proposed, and the local community's response to this process. We have conceptualised the public consultation process as a process of risk communication. We have drawn on theories of risk communication as an analytical focus, including the work of Wynne, to examine the ways in which the public responded to the consultation process.

## 2 Methods

Documentary analysis was used to establish background details about the proposed service changes and the consultation process. This included drivers for change; how the reorganisation was governed and developed; as well as the methods used to involve stakeholders in the decision-making process.

In order to explore the factors that influenced the public response to the consultation process, detailed, individual data were required. These were gathered in one to one interviews – an approach which permits the in-depth exploration of each participant's preferences, motivations and decisions [14].

### 2.1 Study context

Participants were all residents in an urban area of England referred to as ‘Greenville’. At the time, a public consultation was taking place locally about consolidating a range of hospital services on fewer sites, including emergency care. If the proposals went ahead, an urgent care centre for minor injuries and ailments would replace the Emergency Department (ED) at the local district general hospital, ‘Greenville Hospital.’ Residents would be required to travel to another hospital in the area, if they needed to access the services of a full ED.

### 2.2 Documentary analysis

Documentation regarding the Greenville proposals and the public consultation process was collected, principally via the dedicated website and online archive set up by the commissioners proposing the changes. This included strategy documents; records of public meetings and other events; the proposals for change; documentation about the governance of the reconfigurations (such as meeting minutes); as well as local media coverage. Documentation was collected over the lifespan of the study. Qualitative content analysis was carried out, using the systematic method set out by Kohlbacher and others [15].

### 2.3 Semi-structured interviews

#### 2.3.1 Interviewees

Interviews were carried out with individuals from a range of groups, including those likely to attend the ED (older people and parents of young children) [16]; as well as patient representatives. The latter group included members of formal local patient involvement groups and individuals campaigning against the closure of services on behalf of patients. Participants’ demographic characteristics are outlined in Table 1.

Face-to-face semi-structured interviews took place between August 2012 and November 2012, usually at the interviewee's home. Interviews typically lasted between 45 min and 1 h and were recorded and transcribed for analysis. Interviewees were anonymised and details identifying the sites were removed. Participant study numbers are used here to set quotes in context.

#### 2.3.2 Interview design and analysis

A thematic analysis of the interview data was carried out, combining inductive and deductive approaches, using theories about risk communication as an analytic focus, whilst at the same time allowing novel themes to emerge direct from the data [17]. The topic guide for the interviews and the initial broad headings used for analysis both drew on empirical literature regarding the reconfiguration process and conceptual literature about risk communication [18,19]. Pre-defined themes, derived from both literatures, included public engagement, drivers of change, local emergency services and hospital reconfiguration proposals. These themes were expanded and refined inductively and new themes were added to the initial framework [14].

A sample of the transcripts was read by the full research team to identify and agree key themes, after which one researcher coded the interview transcripts. N-Vivo (version 10) was used to manage the analysis.

## 3 Results

### 3.1 Consultation methods

The public consultation in Greenville took place prior to April 2013, when local clinical commissioning groups (CCGs) in England assumed responsibility for purchasing NHS care from primary care trusts (PCTs). A consultation document formed the basis of the process and included questions for members of the public to respond to in writing. It described how the proposed service changes were developed by the local CCGs, made up of GPs representing the PCTs, working with ‘hospital doctors, nurse leaders, providers of community care, social services, patient and volunteer groups and charities.’ In the document, the commissioners set out a range of reasons why they believed local services ‘needed to change in order to improve quality.’ As noted, the proposed changes included consolidating local emergency services on fewer sites. Consequently, in the future, only some of the hospitals in the area would provide a full ED service, emergency surgery, maternity and inpatient paediatric services. Their preferred option for Greenville was to downgrade services on that site, so that the hospital would be left with an urgent care centre, rather than a full emergency service. Most local residents would be expected to travel to the ED at one of the remaining hospitals closest to Greenville.

In all the documentation, particular emphasis was placed on the fact that the plans had been developed by local clinicians. Many of these individuals had worked in the area for a long time, and thus it was argued that they understood the local health care economy very well. The documentation also explained that a range of challenges threatened to impact the delivery of patient care locally, including a growing and ageing population; insufficient numbers of specialists in hospitals to provide round the clock care; inadequate NHS facilities; and increasing financial pressures on the NHS. Consequently, the commissioners sought public views about a range of options aimed at consolidating care on few sites, to make better use of staff expertise, buildings and funds.

Documentation was distributed across the area to GP practices, libraries, hospitals and other health sites, pharmacies, patient groups and local authority offices. The main consultation document was supplemented with a number of other publications, including factsheets about changes to specific services; answers to frequently asked questions; and a public letter outlining the support of senior local clinicians. A dedicated website was created and advertisements were placed in local papers across the affected area and neighbouring regions.

The commissioners also organised a number of public events, including focus groups, roadshows, meetings in hospitals and GP events, as well as public meetings and debates, with the aim of involving ‘as many people and communities’ in the affected area as possible. As well as registering their views at the events, local residents could respond to the consultation in a number of ways, including an online or paper response form. A number of petitions were also submitted by email and post. Paper documentation was distributed in a range of languages. Responses to the consultation were collated and analysed by a large market research company. A final engagement event was then held to present the consultation findings to local stakeholders and gather views about further issues that should be taken into account going forward. Subsequently, a joint committee of local PCTs made a final decision about the proposals.

### 3.2 Public involvement in the consultation process

In terms of involvement in the public consultation process, there were three distinct groups of participants. The first group, whom we refer to as the ‘campaigners against change’ (*n* = 6), strongly opposed the proposals. These individuals had all been recruited to the study as patient representatives and had all sought to express their views via the consultation process. However, their opposition had also led them to get involved in a range of activities above and beyond this formal process. This included arranging their own meetings, campaigning in the street, collecting petition signatures and assisting others to complete the consultation documents. In addition, interviewees in this group all expressed a strong belief that the state should provide communities with a comprehensive local health service.

The second group, in contrast, included two participants who were broadly in favour of the proposed changes in Greenville. We have termed this group ‘non-campaigners in favour of change.’ One of the two, an older interviewee, had previously worked closely with the medical profession; the other was a member of a patient involvement group. Both had engaged with the consultation process principally by attending the public events.

We have termed the third group, which includes all the remaining participants, ‘non-campaigners against change’ (*n* = 12). In this group, most were concerned about the safety implications of having to travel further for care in future. Their opposition was underpinned by an implicit belief that timely access to hospital in an emergency is directly associated with better outcomes [20]. However, only three of the participants in this group (two parent participants; one older person) reported that they had completed the official consultation document, or attended any of the public events. The remaining nine participants had not engaged with the consultation process at all.

In summary, all the ‘campaigners against change’ and the ‘non-campaigners in favour of change’ had registered their views via the consultation process, either by responding to the questions set out in the consultation document or by attending one of the public engagement events. In contrast, only one third of the ‘non-campaigners against change’ reported taking part. Interviewees who had participated in the consultation form the focus of this paper. There were two major factors that influenced the way in which these individuals responded to the process: the behaviour of the commissioners leading the exercise; and the methods used to elicit public feedback. The impact of each of these factors is now described in turn, before we consider their consequences.

### 3.3 Impact of commissioner approaches to consultation

As we have described, the commissioners emphasised that the plans had been developed by local clinicians, many of whom had worked in the area for a long time. However, there was little sense that many of the participants acknowledged that the proposals were clinically-led, or considered this to be important. Most simply referred to the decision-makers behind the plans as an anonymous ‘them.’

In contrast, many of the ‘campaigners against change’ had engaged with the commissioners directly. A local clinical leader represented the public face of the reconfiguration for many in this group, as he had personally met with a number of community groups to discuss the proposals. Commissioners are encouraged to use an ‘expert’ to present the case for change, usually a senior clinician, whose view – it is thought – will carry weight with the community [21]. However, many participants in Greenville remained sceptical about the proposals and simply did not believe the claims put forward by the commissioners, even though the clinical leader had visited patient groups ‘on numerous occasions’, as one put it (Patient Representative 6).

One reason for this scepticism was that the local community did not believe that the commissioners had considered issues of relevance to them when the proposals were first developed. Referring to the clinical leader fronting the proposals, one participant said:

I think he's probably a very clever clinician, but this is not just about clinical factors. This is about a much bigger issue of logistics, of infrastructure, of access. I mean I’m sure he's a very clever man, but he's looking at it in a very cold, clinical [way]. (Patient Representative 7)

This was compounded by the view held by many, that the proposals were aimed principally at cutting costs, rather than improving patient care – a perspective that is often shared by members of the public when such changes are proposed [1]. In fact some disputed the claim that the plans would improve care at all:

This is all to do with the political cuts. Cuts. This has nothing to do with trying to improve A&E. NHS. It's not going to be better. (Patient Representative 2)

Later in the consultation process, the commissioners organised several open meetings to facilitate the engagement of the community with the plans. However, their behaviour at the meetings had created the impression for some attendees that the public's concerns were trivial and irrelevant:

The people running [the meetings] have been somewhat arrogant in the way they’ve talked to clients. Obviously that doesn’t help. I mean you need genuine consultation, with the professionals prepared to listen, not dismissing arguments as trivial. (Older Person 3)

The public meetings were held at prominent venues, where refreshments were provided. This also impacted on the perception that the meetings ‘were not very genuine’ (Patient Representative 9) and that there was ‘some soft soaping going on.’ (Older Person 2)

It was obvious that they were trying to impress people, because lunch was laid on. Because of that, there was a long queue to get in, and I think maybe people came just for the lunch, I don’t know. If people are giving you lunch instead of the facts, they’ve already decided and they’re trying to make it softer for you. (Older Person 2)

At the same time, whilst some commissioners sought to engage with the local community, others were perceived to have failed to engage adequately. This also influenced participants’ perceptions of the process:

I want to feel that I can trust the people who are making those [decisions]. It's about trust. These bloody meetings I went to, the person who never turned up was the woman who's going to run health care in Greenville, the clinical commissioning group. She never turned up at the public meeting at the Town Hall, she never turned up at a volunteers’ meeting that I got up at seven in the morning to go to. She just never turned up. (Patient Representative 7)

The commissioner's failure to attend the public meetings and engage in discussion led this participant to question whether or not the individuals putting forward the proposals could be trusted. On the other hand, the behaviour of commissioners who did meet with the local community raised questions for some participants about whether the factors most relevant to the public, such as access, were taken into account in the decision-making process or had been dismissed as trivial.

### 3.4 Impact of consultation methods

Alongside the public meetings, the local community were invited to put forward their views about the proposed changes by responding to a series of questions set out in the consultation document. This process of collecting feedback was the second key factor that influenced those participants who took part in the consultation exercise. This related to both the volume of documentation provided by the commissioners, including the length of the feedback questionnaire, as well as the way in which the consultation questions were posed.

The consultation document itself was over 80 pages long, and residents were asked to respond to around 30 questions. Participants spoke about ‘a massive document’ (Parent 3) and ‘a massive questionnaire.’ (Older Person 3) For some, the length of the document represented a barrier to them responding:

It's like they’re trying to mess with your heads. I don’t know how many pages it is – I have two kids, I want to take part, but how long is it going to take me? They’re making it too long. It's just unnecessary. (Parent 4)

The ‘campaigners against change’ also expressed concern that, despite its length and the pledges to improve care, the document was not transparent about the implications of the proposals for patients. This particularly related to the possible closure of local services, such as the emergency and maternity departments. Patient Representative 5, a member of a patient involvement group, described the approach of the commissioners in compiling the consultation document as ‘deliberate obfuscation.’ She added:

It was outrageously complex and it, I mean it was just… It was not the sort of thing that you put before the general public. (Patient Representative 5)

Equally, the phrasing of the questions was perceived by some to have excluded opportunities for the public to express disagreement with the plans:

[There was] no option to say ‘actually I fundamentally disagree with all those proposals’ so we just had to write all over our forms we strongly disagree and we think that you should keep our hospitals exactly as they are. (Patient Representative 9)

Other participants perceived the consultation questions to be leading respondents into agreeing with the proposals:

The way the questions were posed, led you into agreeing… I mean, it was a very, very ill… No, not ill conceived – they did exactly what they wanted – but it was not a consultation as I understand it, which is about getting agreement around the issue and then looking at how it could be tackled. It was very much, ‘our preferred option is this, but if you want to vote for Greenville Hospital, you can close [another hospital instead],’ so it was pitting communities against communities and hospitals against hospitals and things like that. And you know, it all started off with, you know, ‘would you like better services and more services?’ And they all looked sort of, ‘oh yeah that's a good idea’ and then you get to it, ‘well of course you’ve got to do what we say then.’ (Patient Representative 8)

### 3.5 Consequences

Disenchantment with the consultation process led many of those who participated to question the motivations of the commissioners. Some argued that the changes were not aimed at improving care, but were instead part of a wider programme of financial cuts. Others felt that the commissioners were deliberately misleading the public about the true implications of the changes. By constructing a narrative that depicted the commissioners as driven by hidden motives, those opposed to the proposals were able to dismiss the legitimacy of the commissioners’ arguments, further fuelling the perception that they were untrustworthy.

As well as undermining trust, Wynne argues that, in the context of risk communication, a failure to connect with the local community will result in conflict and dissonance [19]. In non-health care settings, one way in which this has been shown to manifest is in lay people seeking to challenge expert prediction and advice [22]. In the light of public concerns about having to travel further for care, several campaigners in Greenville sought to demonstrate the implications of the proposed changes. For example, some took public transport to the nearest alternative hospital, to highlight the challenges patients would face in the future. Others had scrutinised the data underpinning the case for change, including the pre-consultation business case, and had submitted freedom of information requests for data about Greenville Hospital, as ‘part of building up evidence’ to oppose the changes. (Patient Representative 8)

The second way in which the campaigners sought to challenge the views of the commissioners was to invoke the views of other medical professionals to support their case, arguing that local clinicians were largely opposed to the proposals. One participant, a member of a patient involvement group, described her encounters with local doctors at the public meetings:

We’ve spoken to a lot of the doctors – consultants – who work [at Greenville Hospital], going to these various meetings and they are very concerned about [the proposals]… I mean they have been very dedicated coming to quite a lot of these public meetings. (Patient Representative 5)

By framing the commissioners as heartless bureaucrats, intent on pushing through the changes, the campaigners were able to bracket them as ‘other’ and mark out a clear contrast with their own trusted clinicians, who in many cases were seen to be on the side of the local community and opposed to the changes.

## 4 Discussion

The public engagement methods used by commissioners in Greenville reflect the approach currently advocated by NHS guidance, which states that ‘change must be clinically-led and underpinned by a clear clinical evidence base’ [23]. As we have outlined, such an approach appears to assume that if local communities are presented with the ‘right evidence’ they will be convinced of the need to change [6]. This reflects a traditional psychometric approach to risk communication, which suggests that the public have exaggerated fears because they hold insufficient or incorrect information about an issue [24]. Consequently, it is assumed that efforts to reduce the ‘deficit’ in their understanding, for example by providing additional information, will lessen their concerns [25]. However, this model has received widespread criticism in non-health care settings [22]. It is said to be underpinned by several ‘flawed assumptions.’ These include the belief that medical science alone can provide objective truths; that scientific and technical experts are the only sources of valid and rational information; and finally, that the public is a passive receiver of risk information [26]. The accounts of interviewees suggest that all of these were in evidence in Greenville. The participants who were involved in the consultation process, including the campaigners and the members of the wider public who took part, believed that their concerns were considered irrelevant and trivial by the commissioners, who focussed principally on communicating the clinical rationale for change.

Trust and credibility are also widely acknowledged in the risk communication literature as major factors influencing the uptake and understanding of scientific messages [19]. However, this literature also demonstrates that trust is difficult to create and easy to lose; once lost, it is difficult to regain [27]. As we have shown, trust played a pivotal role in the public's response to the engagement process in Greenville, particularly for those who were actively involved. Drawing on the deficit model, the commissioners had sought to build trust by emphasising that the plans had been drawn up by local clinicians. They also sought to allay the community's concerns via public engagement events and publication of a document which set out the case for change. However, the community's belief that the proposals were driven by financial motives, together with the commissioners’ actions, led to a perception that the commissioners could not be trusted. This concern then drove the campaigners to challenge the case for change and invoke the contrasting views of other local doctors, which further served to fuel their frustration and perpetuate a cycle of mistrust.

This study provides, for the first time, a detailed exploration of the way in which local communities may respond to processes designed to engage them in decisions about consolidating local hospital services on fewer sites. Using theories of risk communication as an analytical focus, we have been able to offer a range of new and important insights regarding the potential impact of the engagement approach advocated by policy. In particular, we have been able to suggest several reasons why strategies that seek to ‘educate’ the public may not work as expected [4]. We have also demonstrated that, when changes to local services are proposed, there may be a spectrum of opinions across the affected community, including some individuals in favour of change. Although there were relatively small numbers of participants in some sub-groups, such as the group in favour of change, our work supports Parkinson's argument that the views of those campaigning against reconfiguration proposals, who are most often heard in debate, may not be representative of the community at large [28]. Nevertheless, our findings are based on a single reconfiguration, taking place in an urban area. They may therefore be less relevant in other settings, for example rural areas, where access may be an even bigger concern for local residents. Our findings are also based on a relatively small number of interviews, which represents another limitation of the study, together with the relatively small numbers in some sub-groups. Each community and reconfiguration proposal is also different [1] and different approaches to public consultation may be more or less successful in different areas. However, the concerns raised in Greenville echo those raised previously by residents in other parts of the country [1,29]. Our specific goal was to explore factors that influence the way members of the public respond to consultation processes. We did not interview the commissioners who were conducting the consultation. However, the perspective of this group is clearly important in seeking to develop recommendations about how to improve the process. Additional qualitative research involving those running consultations would provide important insights about their goals; the underlying rationale for their chosen approach; and their perceptions of facilitators and barriers to effective engagement.

The reconfiguration of stroke services in London has been cited as an example of good public engagement practice [21]. Some argue that the changes in London were implemented with relatively little opposition because clinical leaders played a prominent role in articulating the rationale behind the proposals [21]. However, clinical leadership and a detailed explanation of the case for change failed to change the opinion of many participants in Greenville. As a possible explanation for this difference, other studies have observed that the strength of local opposition is related to the content of the reconfiguration proposals, particularly the extent to which services are being withdrawn. Where reconfiguration is perceived as a ‘downgrading’ of service provision, there is more conflict [6]. Crucially, in London, although some hospitals lost their acute stroke services, the reorganisation did not result in changes to ED service provision, or other services which may be particularly important for the public, such as maternity and paediatrics [6]. It is possible that the relative lack of conflict in London was at least partly due to this. In contrast, in Greenville, clinical leadership of the proposals appears to have been insufficient to change the opinion of most study participants when emergency services were being withdrawn. Indeed, the literature on environmental risks strongly suggests that this approach is not sufficient for the provision of credible scientific advice [18].

In Figure 1, we cited the Independent Reconfiguration Panel's assessment of key shortcomings in local consultation processes [1]. We have used this assessment to frame our recommendations. Our findings offer important insights into why these issues create concern amongst local communities; how communication might be improved; and where additional research would be valuable. First, however, it is important to set this in context. Spurgeon et al. assert that within the setting of the current English NHS, there is a ‘likelihood that conflict over hospital reconfiguration will persist’ [4]. Our work outlines some reasons why this may be the case. However, whilst reconfiguration proposals involve the withdrawal of services, we suggest that there can be no ‘magic bullet’ that will lead to their smooth acceptance. This is not least because members of the public appear to interpret the implications of reconfiguration plans in a different way to commissioners and clinicians, particularly with respect to quality and safety [20]. Nevertheless, the IRP note that in many reconfiguration situations, the consultation process is flawed because ‘the clinical case has not been convincingly described or promoted’ [1]. In contrast, in Greenville, the case for change was comprehensively outlined, but we have also explained why this approach may also fail to convince the public. Instead, the Kings Fund suggest that a ‘conversation’ needs to take place between clinicians and populations [12]. Our findings support this and we also suggest that this dialogue should take into account the way in which risk is interpreted by the public in this context, drawing from other related areas, such as health protection [22]. We recommend that further qualitative research is undertaken to identify practical ways in which consultation processes could be improved, for example, patient and public preferences for involvement in decision-making; views of change leaders regarding obstacles to engagement and how they might be overcome; and how these stakeholder groups view recommendations to improve existing approaches to involvement. The IRP also highlight the problem of ‘important content missing from reconfiguration plans and limited methods of conveying information.’ In Greenville, a wealth of information was provided but participants argued that the consultation information did not clearly state what would happen to some services, fuelling mistrust. In future, commissioners must ensure that local communities are ‘clear what services will be provided, where and how they will access them’ [1]. Crucially, the public in Greenville also did not believe that the questionnaire gave them opportunity to oppose the proposals. Finally, the IRP also highlight ‘inadequate attention given to the responses during and after the consultation’ [1]. The panel advise that commissioners should take time to consider responses. We would go further to recommend that decision-makers should explicitly acknowledge and proactively address public responses to the consultation, as part of their ‘conversation’ with local communities. Our exploration of the ways in which the public respond to both the method of consultation, and the content of reconfiguration proposals [20], offers commissioners new insights into why the public may respond in certain ways – for example around the safety implications of service reorganisation – which may help those planning reconfigurations in future.

## 5 Conclusion

Our study challenges the assumption that evidence can be used to persuade communities to accommodate service reorganisations which may compromise timely access [6]. Drawing on theories of risk communication has led to new knowledge about the potential impact of methods currently used to engage the public. In particular, rather than improving trust, the approach advocated by policy [1,3,12] may in fact have the opposite effect and contribute to public opposition. This is partly because it assumes that technical experts are the only sources of valid and rational advice, whilst the public are merely passive receivers of risk information [26]. Current recommendations seek to improve the consultation process by providing the public with more details about the case for change. However, this approach may fuel resistance amongst the local community, if commissioners continue to regard medical knowledge as pre-eminent and fail to acknowledge residents’ local knowledge and perspectives on health care.

## Figures and Tables

**Table 1:** Participant demographic characteristics.

	Number of participants	Number of females	Number from Black and Minority Ethnic Groups	Age range
Parents	5	5	1	28–40
Older participants	6	2	0	65–85
Patient representatives	9	4	2	60–76
Total	20	11	3	28–85
